# A new multidrug‐resistant enterotoxigenic *Escherichia coli* pulsed‐field gel electrophoresis cluster associated with enrofloxacin non‐susceptibility in diseased pigs

**DOI:** 10.1111/jam.14816

**Published:** 2020-08-25

**Authors:** M. de Lagarde, G. Vanier, G. Desmarais, H‐R. Kohan‐Ghadr, J. Arsenault, J.M. Fairbrother

**Affiliations:** ^1^ OIE Reference Laboratory for Escherichia coli Faculty of Veterinary Medicine Université de Montréal Saint‐Hyacinthe QC Canada; ^2^ Department of Obstetrics, Gynecology and Reproductive Biology College of Human Medicine Michigan State University Grand Rapids MI USA; ^3^ Swine and Poultry Infectious Research Center (CRIPA‐FQRNT), and Groupe de recherche en épidémiologie des zoonoses et santé publique (GREZOSP) Faculty of Veterinary Medicine Université de Montréal Saint‐Hyacinthe QC Canada

**Keywords:** antimicrobial resistance, clonal complex, *Escherichia coli*, ETEC, fluoroquinolones non‐susceptibility, pigs

## Abstract

**Aims:**

To describe the temporal trends in *Escherichia coli* pathotypes and antimicrobial resistance detected in isolates from diseased‐pig cases submitted to the EcL from 2008 to 2016, in Quebec, Canada, and to investigate the presence of spatiotemporal and phylogenetic clusters.

**Methods and Results:**

Detection of 12 genes coding for virulence factors in pathogenic *E. coli* in pigs by PCR and antimicrobial resistance standard disc diffusion assay were performed. Demographic and clinical data were entered in the Animal Pathogenic and Zoonotic *E. coli* (APZEC) database. ETEC:F4 was the most prevalent pathovirotype among the 3773 cases submitted. The LT:STb:F4 virotype was predominant until 2014, then was overtaken by the LT:STb:STa:F4 virotype. More than 90% of the ETEC:F4 isolates were multidrug resistant. A spatiotemporal cluster of LT:STb:STa:F4 isolates non‐susceptible to enrofloxacin was detected between 4/2015 and 9/2016. Pulsed‐field gel electrophoresis analysis of 137 ETEC:F4 isolates revealed the presence of a cluster composed mainly of LT:STb:STa:F4 isolates non‐susceptible to enrofloxacin.

**Conclusions:**

The APZEC database was useful to highlight temporal trends in *E. coli* pathotypes. A high‐risk ETEC:F4 clone might disseminate in the pig population in Quebec since 2015.

**Significance and Impact of the Study:**

Surveillance is crucial to identify new clones and develop control strategies.

## Introduction

With more than seven million of pigs marketed in 2020, the Quebec porcine industry is the largest producer of pig meat in Canada, representing 30% of the total Canadian production. *Escherichia coli* is an important cause of a wide range of diseases in pigs, including neonatal and postweaning diarrhoea (respectively ND and PWD) and oedema disease, resulting in significant economic losses worldwide due to morbidity associated with excessive weight loss and mortality (Gyles *et al*. 2010). Strains of the most important pathotype in pigs, the enterotoxigenic *E. coli* (ETEC), produce one or more enterotoxins, which act on the intestinal epithelial cells to induce the secretion of water and electrolytes into the intestinal lumen, causing diarrhoea (Gyles *et al*. 2010). The most important enterotoxins, which define ETEC, are the heat‐labile toxin LT and the heat‐stable toxins STa and STb. ETEC must be able to adhere to and colonize the intestinal mucosa to permit the release of sufficient levels of enterotoxin to result in the development of diarrhoea. This adherence is mediated by hair‐like structures on the bacterial surface, called fimbriae or pili. ETEC associated with neonatal diarrhoea may produce one or more of the fimbriae F4 (K88), F5 (K99), F6 (897P), and F41 (Gyles *et al*. 2010). ETEC associated with PWD most commonly produce F4 (K88) or F18 (F107) fimbriae (Dubreuil *et al*. 2016). In swine, the neonatal and post‐weaning periods are critical for ETEC infections.

A second pathotype found in pigs with diarrhoea is known as enteropathogenic *E. coli* (EPEC). These bacteria cause typical attaching and effacing lesions and possess a variant of the EPEC attaching effacing factor Eae or Intimin (Gyles *et al*. 2010; Zimmerman *et al*. 2019).

Shiga toxin producing *E. coli* (STEC) produce one or more of a family of cytotoxins which are known collectively as Shiga toxins (Stx) or verotoxins (VT). In pigs, the most important STEC are those that cause oedema disease. These strains produce the toxin variant Stx2e (VT2e) and the fimbriae F18. Certain isolates produce both Stx2e and enterotoxins, as well as the fimbriae F18. These isolates are associated more with PWD than oedema disease (Gyles *et al*. 2010). Such isolates are designated as ETEC/STEC hybrids (Nyholm *et al*. 2015; Dubreuil *et al*. 2016).

Identification of pathogenic *E. coli* based on the presence of virulence genes has permitted a more accurate diagnosis of diseases caused by these bacteria although there are few reports on the organization of such data to monitor the trends in *E. coli* infections in diseased pigs. The Animal Pathogenic and Zoonotic *E. coli* (APZEC) database (apzec.ca) gathers information from the OIE Reference Laboratory for *Escherichia coli* (EcL), Canada. This integrated tool, designed by the EcL, permits the monitoring of the temporal trends of the different pathogenic *E. coli* detected at the laboratory, since 1990. Thus, in swine, in Quebec, most pathogenic *E. coli* detected in ND and PWD cases belong to one of the various virotypes comprising the ETEC pathotype and possess F4, which, collectively, has been designated the ETEC:F4 pathovirotype. Until 1995, isolates belonged predominantly to the virotype LT:STb:F4 (data not shown). Between 1995 and 2005, two clonal lineages of virotype LT:STb:STa:F4 emerged and were associated with an increase of severity of PWD (Noamani *et al*. 2003). From 2006 to 2013, PWD was mostly associated with two other clonal lineages from the virotype LT:STb:F4 (Jahanbakhsh *et al*. 2016). Hence, predominant virotypes of the ETEC:F4 isolates detected in pigs with diarrhoea in Quebec have varied with time.

Since 2014, the number of diarrhoea cases submitted to the EcL for detection of pathogenic *E. coli* in pigs has increased in Quebec. This is of concern for porcine health, as these cases were often associated with fluoroquinolone (especially enrofloxacin, Baytril®; Bayer, Shawnee Mission, KS) non‐susceptible *E. coli* isolates. The therapeutic use of quinolones in porcine medicine seems to have resulted in the selection of resistant isolates (Hoelzer *et al*. 2017). The presence of this resistance is of major concern for several reasons. First, quinolones are classified as very highly important in human medicine by the WHO and Health Canada (Health Canada 2009; World Health Organization 2019). Second, their use has been associated with the emergence of ‘high‐risk’ clones such as the *E. coli* ST131 in the human population. ‘High‐risk’ clones are defined as the progeny of one bacterium through asexual multiplication having a global distribution and being associated with multidrug resistance and high virulence (Mathers *et al*. 2015). In addition, they have a great capacity to disseminate from host to host and appear to be very successful vehicles for mobile genetic elements (MGE) (such as plasmids and integrons). In ETEC, it is recognized that virulence factors are usually plasmid mediated, and that some of the circulating plasmids also carry antimicrobial resistance genes (Shepard *et al*. 2012), such as those encoding β‐lactamases which also have a high importance in human medicine. Therefore, the presence of one or several high‐risk clone(s) in the porcine population would present a potential risk for public and porcine health.

Based on our observations, we hypothesized that one or more new potential ‘high‐risk’ clonal lineage(s) have emerged in the porcine population, in Quebec, in recent years.

The main objective of this study was to describe the temporal trends in virotypes detected in diseased pigs in Quebec from 2008 to 2016 using the APZEC database and report the temporal trends of fluoroquinolone and multidrug resistance in these isolates. Additional aims were to investigate the presence of a spatiotemporal cluster of the ETEC isolates of the virotype LT:STb:STa:F4, associated with fluoroquinolone resistance, and to assess a potential phylogenetic relationship between ETEC:F4 isolates, based on their pulse‐field gel electrophoresis (PFGE) profile, to identify the presence of one or several potential ‘high‐risk’ clones in the porcine population.

## Materials and methods

### Bacterial isolates and virulence gene detection

**Figure 1:**
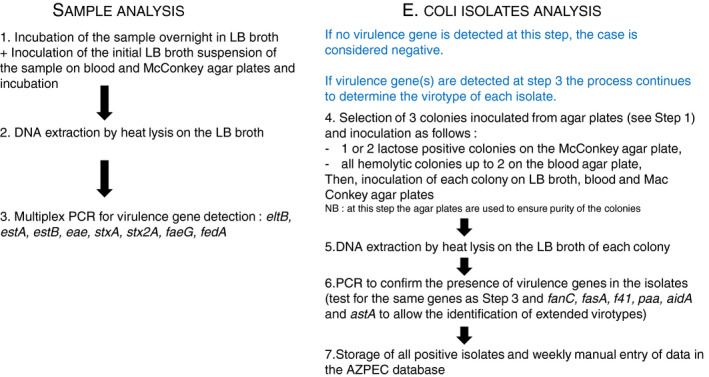
Outline of the EcL approach for the detection of the virulence genes which define the *Escherichia coli* pathotypes commonly found in pigs. LB = Luria–Bertani broth. [Colour figure can be viewed at wileyonlinelibrary.com]

Primary *E. coli* cultures were obtained from the intestinal contents or faeces of pigs with intestinal disease submitted to the Laboratoire de Santé Animal (LSA) of the Ministère de l'Agriculture, des Pêcheries et de l'Alimentation du Québec (MAPAQ) or the Diagnostic Service of the Faculté de médecine vétérinaire (FMV) of the Université de Montréal, or from faecal swabs collected by veterinary practitioners from diseased pigs and submitted to the FMV. The samples had originally been plated on both Tryptone soya agar plates (ThermoFischer Scientific, Oxoid Company, Ontario, Canada) with 5% sheep blood and MacConkey agar plates (Becton Dickinson, Franklin Lakes, NJ) at 37°C. Then, samples were transferred to the EcL and enriched overnight in Luria‐Bertani (LB; Difco, Mississauga, ON, Canada) broth (Fig. 1). Next, DNA was extracted from the LB culture by heat lysis, as previously described (Maluta *et al*. 2014). Multiplex PCR was carried out at the EcL for detection of the virulence genes which define the *E. coli* pathotypes commonly found in pigs: ETEC (*eltB, estA,* and *estB*), EPEC (*eae*), STEC (*stxA, stx2A*), and *faeG* and *fedA* genes encoding the fimbrial adhesins F4 (K88) and F18 respectively. When one or more pathotype‐defining genes were identified in the sample, three colonies were selected, among lactose‐positive and haemolytic colonies and resubmitted to culture enrichment in LB broth to obtain pure cultures. The three isolates were examined by multiplex PCR for the presence of the virulence genes which define the *E. coli* pathotypes commonly found in pigs, as above. Pathotype positive isolates were further examined by multiplex PCR for the presence of the genes fanC (K99), fasA (F6), f41 (F41), paa (Paa), aidA (AIDA) and astA (EAST1), to determine the extended virotype. An ETEC or STEC isolate was considered as a pathogenic agent if it possessed a fimbrial adhesin gene, or as a possible pathogenic agent if it did not possess any of these fimbrial adhesin genes. EcL protocols and the APZEC database are available online on http://www.apzec.ca/en/Protocols and www.apzec.ca respectively.

### Antimicrobial susceptibility testing

One ETEC:F4 isolate per virotype and per case (see definition in next section) was randomly selected for antimicrobial susceptibility testing. These isolates were tested by the LSA (MAPAQ) or the Service Diagnostic de la Faculté de Médecine Vétérinaire for susceptibility to 10 antimicrobial agents using the disc diffusion (Kirby‐Bauer) assay as previously described (Jahanbakhsh *et al*. 2015). The following antimicrobial disks were used: apramycin (15 μg), neomycin (30 μg), gentamicin (10 μg), streptomycin (10 μg), ceftiofur (30 μg), enrofloxacin (5 μg), trimethoprim‐sulfamethoxazole (1·25/23·75 μg), ampicillin (10 μg), florfenicol (30 μg) and tetracycline (30 μg). The generic *E. coli* ATCC® 25922 served as control. Veterinary CLSI 2015 clinical breakpoints (defined for animal pathogens) were used when available (CLSI 2015a); otherwise, CLSI 2015 clinical breakpoints (defined for human pathogens) were used (CLSI 2015b). Isolates were considered to be multidrug resistant (MDR) if they were non‐susceptible (resistant or intermediate) to at least one antimicrobial in three or more classes of antimicrobials tested, as previously described (Magiorakos *et al*. 2012).

### The APZEC database

The APZEC database is an information system developed in‐house by the authors in the EcL at the Faculté de Médecine Vétérinaire, Université de Montréal. It comprises three major components: first, the back‐end SQL server database, a robust relational database system to store the generated data; second, the front‐end application developed using the Microsoft Visual Basic that provides graphical user interfaces (GUIs) to enter and forward the collected clinical and laboratory data to the back‐end database server; and finally, the web‐based interface (http://www.apzec.ca) for data presentation, built using Visual Basic language and ASP.NET technology that contains Microsoft’s framework and toolsets for building Web‐based applications.

For each isolate, the virulence genes, antimicrobial resistance profile and information on the sampled animal (species, age and clinical signs) were entered weekly in the APZEC database using the front‐end GUI application, resulting in an integrated profile. Since 2013, the localization of sampling site (6‐digit postal code) has also been systematically captured in the database for each isolate.

A ‘case’ was defined as a gathering of samples coming from the same farm on the same day, but not necessarily coming from the same animal. A ‘positive case’ was defined as a case where a pathogenic agent or possible pathogenic agent was detected. A positive case for a specific pathovirotype was defined as a case with detection of one or more isolates from this pathovirotype. As several pathovirotypes may be associated with a case, a case can be positive for multiple pathovirotypes.

### Descriptive statistical analysis

Based on the APZEC‐compiled results, the percentage of positive cases per pathovirotype was calculated per year among all cases from diseased pig samples submitted in Quebec from 2008 to 2016. Subsequently, the number of cases positive for each virotype among ETEC:F4 positive cases was determined per year using the same database. The number of isolates non‐susceptible to enrofloxacin and the number of MDR isolates were presented per year and per virotype. Also, the number of non‐susceptible isolates per antimicrobial among ETEC:F4 isolates was presented per year.

### *Space and time analysis of cases according to the detection of* LT:STb:STa:F4 *isolates non‐susceptible to enrofloxacin*

Enrofloxacin non‐susceptibility was mostly associated with the virotype LT:STb:STa:F4 in our dataset (see results and Fig. 4). Therefore, the Kulldorff space‐time scan statistic was used to assess whether cases with detected LT:STb:STa:F4 isolates non‐susceptible to enrofloxacin were randomly distributed, or if they were clustered in space and/or time (Kulldorff 1997). This test was performed with the software SaTScan 9.4.4 (available at https://www.satscan.org/). This analysis was performed at the case level and was limited to the 2013–2016 period based on availability of the postal code data. The day of sampling was used as the time unit. Each case was geolocated at the centroid of the 6‐digit postal code area of the farm with GeoPinpoint^TM^ Suite (DMTI Spatial Inc 2008). The detection of an LT:STb:STa:F4 isolate non‐susceptible to enrofloxacin in an APZEC case was considered as a case, all other APZEC cases were considered as controls. Therefore, a Bernoulli model was used with statistical significance determined using 999 permutations. The ArcGis10·3.1 software (ESRI) was used to map the results.

### Phylogenetic analysis by pulse‐field gel electrophoresis

A sample of 137 isolates from the LT:STb:STa:F4 or LT:STb:F4 virotype detected between 2013 and 2016 inclusively were randomly selected with probabilities proportional to the number of isolates detected for each year and virotype. These isolates were examined by PFGE using XbaI restriction enzyme as described by Pulsnet (Ribot *et al*. 2006; Vounba *et al*. 2018). PFGE profiles were analysed using bionumerics 6.6 software (Applied Maths, Sint‐Martens‐Latem, Belgium). An additive tree was generated by the unweighted pair‐group method using arithmetic (UPGMA) averages, based on the Dice similarity coefficient (optimization 1%; tolerance 1%). The following definitions were used (Vounba *et al*. 2018): isolates with 95% of similarity were defined as a pulsotype and; isolates with 55% of similarity were defined as a PFGE cluster.

## Results

### Emergence of the virotype LT:STb:STa:F4 throughout the study period was associated with an increase in the proportion of cases with detection of isolates non‐susceptible to enrofloxacin

Samples originating from a total of 3773 porcine cases were submitted to the EcL Laboratory between 2008 and 2016. Overall, pathogenic and possible pathogenic *E. coli* isolates were identified in 1870 (49·6%) cases and 1903 (50·4%) cases were negative. The distribution of cases according to clinical signs is detailed in Table S1.

**Figure 2:**
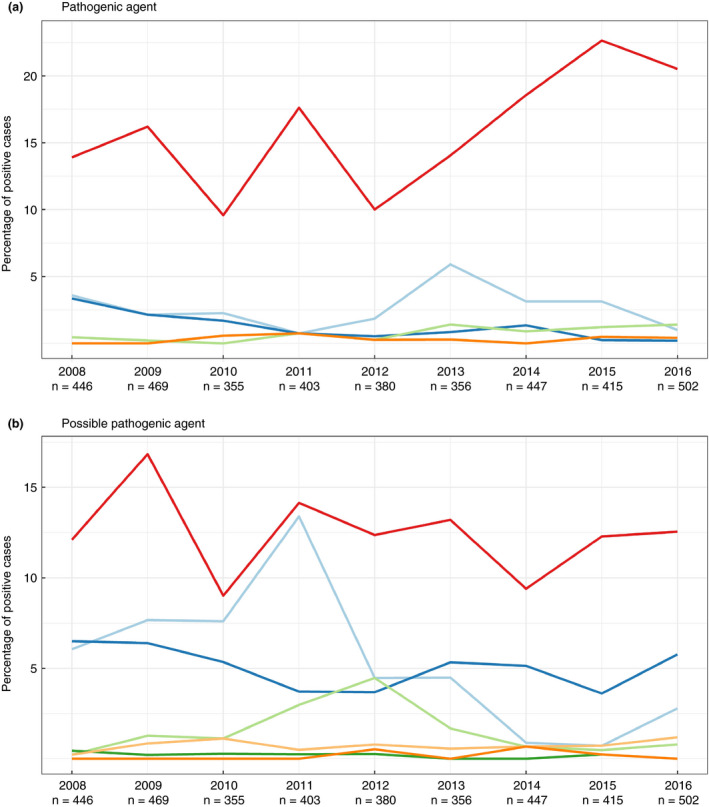
Percentage of positive cases per pathovirotype in diseased pigs in Quebec among all cases submitted to the EcL from 2008 to 2016. The pathovirotypes were classified as pathogenic agents if they carried a colonization factor (F4, F18 or F5) and as possible pathogenic agents if they carried only enterotoxins, shiga toxin or the *eae* gene. Cases are defined as a gathering of samples coming from the same farm on the same day, but not necessarily from the same animal. Undetermined: the sample possesses only fimbrial genes (and could represent a vaccinal strain). During the study period there were 3773 cases and 1870 positive cases, including 89 mixed cases (i.e. several pathovirotypes per case) (Pathovirotype: a: 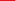 ETEC:F4; 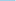 STEC:F18; 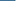 ETEC:F5; 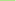 ETEC:F18; 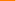 ETEC:STEC:F18; b: 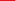 ETEC; 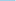 undetermined; 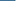 EPEC; 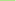 STEC; 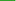 EPEC:STEC; 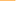 ETEC:STEC; 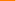 ExPEC). [Colour figure can be viewed at wileyonlinelibrary.com]

Among the pathogenic agents, ETEC:F4 was the predominant pathovirotype identified, with an increase of its detection from 10 to 23% of cases between 2012 and 2015 (Fig. 2). Although less often identified, STEC:F18 was the second most predominant pathovirotype (between 4 and 6% of total cases throughout the study period). ETEC:F18 and ETEC:STEC:F18 were not predominant in Quebec during the study period (detected in <3% of cases).

Among pathotypes considered as possible pathogenic agents, ETEC‐positive cases were found the most frequently, in a relatively stable percentage of cases (between 9 and 16% of cases) each year throughout the study period (Fig. 2). EPEC cases oscillated around 5% each year during the study period and STEC‐positive cases were identified rarely (<2% of the cases) except for the years 2011 and 2012 when almost 5% of the cases were STEC.

**Figure 3:**
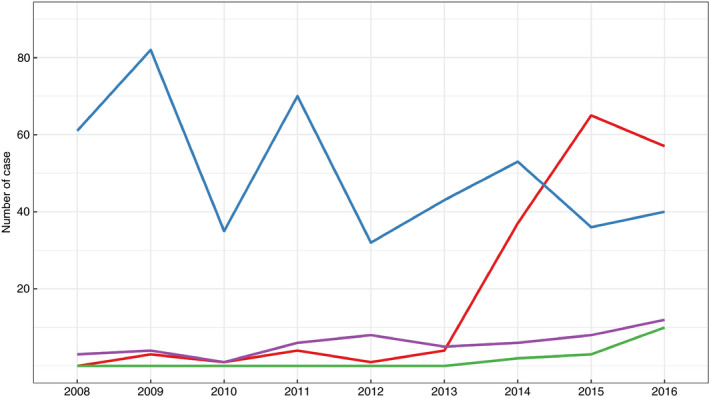
Number of positive cases per virotype in diseased pigs in Quebec among ETEC:F4 cases submitted to the EcL from 2008 to 2016. During the study period there were 611 ETEC:F4 cases, including 81 mixed cases (i.e. several virotypes per case). Miscellaneous ETEC:F4 is a group of isolates carrying other combinations of enterotoxin genes (virotype ETEC:F4: 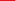 LT:STb:STa; 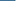 LT:STb; 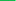 STa:STb; 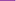 miscellaneous). [Colour figure can be viewed at wileyonlinelibrary.com]

In total, during the study period, 692 ETEC:F4 isolates were identified in 611 cases (16% of all cases). Figure 3 illustrates the temporal trends in the number of cases with the different ETEC:F4 virotypes detected throughout the study period. The virotype LT:STb:STa:F4 was not frequently observed between 2008 and 2013. The number of cases positive for isolates of this virotype increased dramatically in 2014 and 2015. The frequency of cases positive for LT:STb:F4, which indisputably predominated among ETEC:F4‐positive cases before 2014 (with 78 cases representing 95% of cases positive for ETEC:F4 belonging to this virotype), decreased to become less than that of cases positive for LT:STb:STa:F4 after 2014 (between 33 and 35% of cases positive for ETEC:F4 isolates belonging to the LT:STb:F4 virotype in 2015 and 2016). Another virotype, STa:STb:F4, appeared in 2014 and its frequency seems to have increased subsequently.

**Figure 4:**
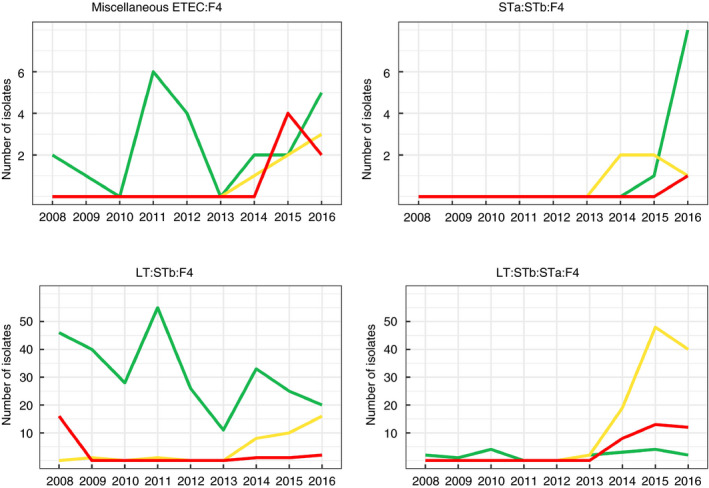
Susceptibility to enrofloxacin in ETEC:F4 isolates from positive cases, classified by virotype, detected in samples from diseased pigs of Quebec submitted to the EcL from 2008 to 2016. Miscellaneous ETEC:F4 is a group of isolates carrying other combinations of enterotoxin genes. During the study period there were 692 ETEC:F4 isolates from 611 cases, including 81 cases with more than one isolate. Only one isolate per virotype was kept for each case (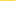 susceptible; 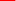 intermediate; 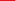 resistant). [Colour figure can be viewed at wileyonlinelibrary.com]

Overall, the proportion of ETEC:F4 isolates non‐susceptible to enrofloxacin increased over the study period (Fig. 4) from 0% (0/64) in 2008 to 69% (77/112) in 2016. Most LT:STb:STa:F4 isolates, since their appearance in 2014, have been non‐susceptible to enrofloxacin. In 2014, few LT:STb:F4 isolates were non‐susceptible to enrofloxacin (19%) with this proportion increasing in subsequent years. In 2016, almost half (45%) of the LT:STb:F4 isolates were non‐susceptible to enrofloxacin. Isolates belonging to the STa:STb:F4 virotype were predominantly sensitive to enrofloxacin (in 2016, 20% of STa:STb:F4 isolates were non‐susceptible to enrofloxacin).

The results for the extended pathovirotypes (*fanC*, *fasA, f41, paa*, *aidA* and *astA)* are available in the supplemental data (Table S2).

### Increase of multidrug resistance in ETEC: F4 isolates in diseased pigs in Quebec from 2008 to 2016

**Figure 5:**
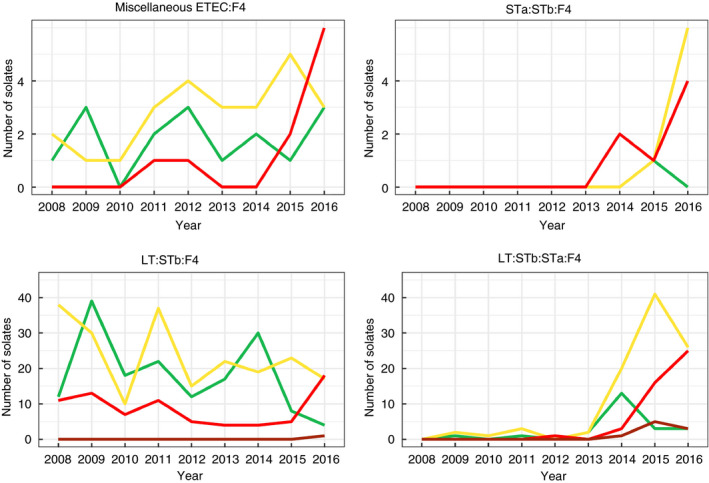
Number of multidrug resistant ETEC:F4 isolates, classified by virotype, detected in samples from diseased pigs of Quebec submitted to the EcL from 2008 to 2016. Miscellaneous ETEC:F4 is a group of isolates carrying all other combination of enterotoxin genes. During the study period there were 692 ETEC:F4 isolates from 611 cases, including 81 cases with more than one isolate. Only one isolate per virotype was kept for each case (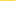 0–2 classes; 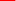 3–4 classes; 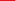 5–6 classes; 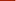 7–8 classes). [Colour figure can be viewed at wileyonlinelibrary.com]

For ETEC:F4 isolates of each virotype, the percentage of isolates non‐susceptible to three classes of antimicrobial and more increased from 80 to 92% (Fig. 5).

Possible extensively resistant isolates (resistant to 7 or 8 of the eight tested antimicrobial classes) appeared in 2015 and 2016 for LT:STb:STa:F4 and LT:STb:F4 virotypes respectively.

Temporal trends of ETEC:F4 isolates non‐susceptible to the nine other antimicrobials tested are described in the Fig. S1.

### *Spatiotemporal cluster of* LT:STb:STa:F4 *non‐susceptible to enrofloxacin occurred at the end of 2015*

**Figure 6:**
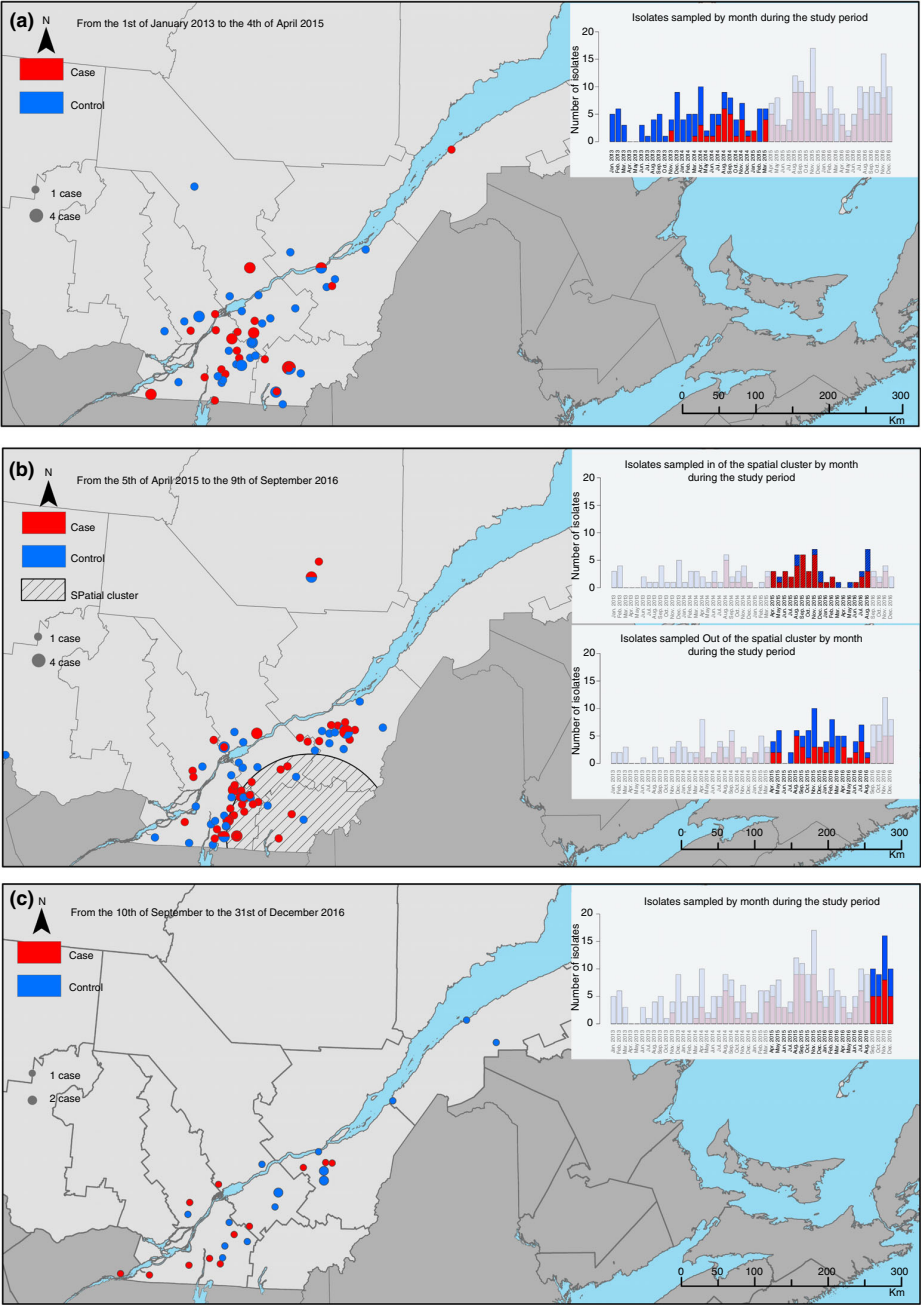
Representation of the spatio‐temporal cluster. Geographical distribution of cases (in red) and control (in blue) in the province of Quebec, Canada, before (a), during (b) and after (c) the spatiotemporal cluster. On each map, the graphic in the upper right indicates the temporal distribution of cases and controls sampled per month during the study period. The highlighted period is the period illustrated on the corresponding map. On the map b, the striped circle indicates the spatial cluster, and the striped bars represent the cases sampled *in* the spatial cluster, whereas the unstriped bars represent the cases sampled *out* of the spatial cluster. The size of the circles indicates the number of cases at this location. In this analysis, a case is defined as an APZEC case positive for an ETEC:F4:LT:STa:STb isolate non‐susceptible to enrofloxacin and a control is any other APZEC case (a: 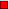 case; 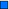 control;  1 case; 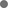 4 cases: b: 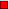 case; 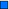 control; 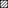 spatial cluster;  1 case; 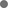 4 cases: c: 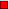 case; 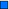 control;  1 case; 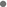 2 cases).

One statistically significant spatiotemporal cluster was detected. The relative risk associated with this cluster was 2·33 (*P*‐value <0·001) meaning that there were 2·33 times more cases (i.e. cases positive for an LT:STb:STa:F4 isolate non‐susceptible to enrofloxacin) than controls in this area during the cluster time frame, which was from the 5 April 2015 to the 9 September 2016. The coordinates of the centre of the cluster were 45·199529 N, 71·500486 W with a radius of 113·8 km, roughly matching the centre of the Montérégie region of the province of Quebec (Fig. 6).

### *PFGE cluster (55% of similarity) of* LT:STb:STa:F4 *non‐susceptible to enrofloxacin predominates since 2015 in Quebec*

**Figure 7:**
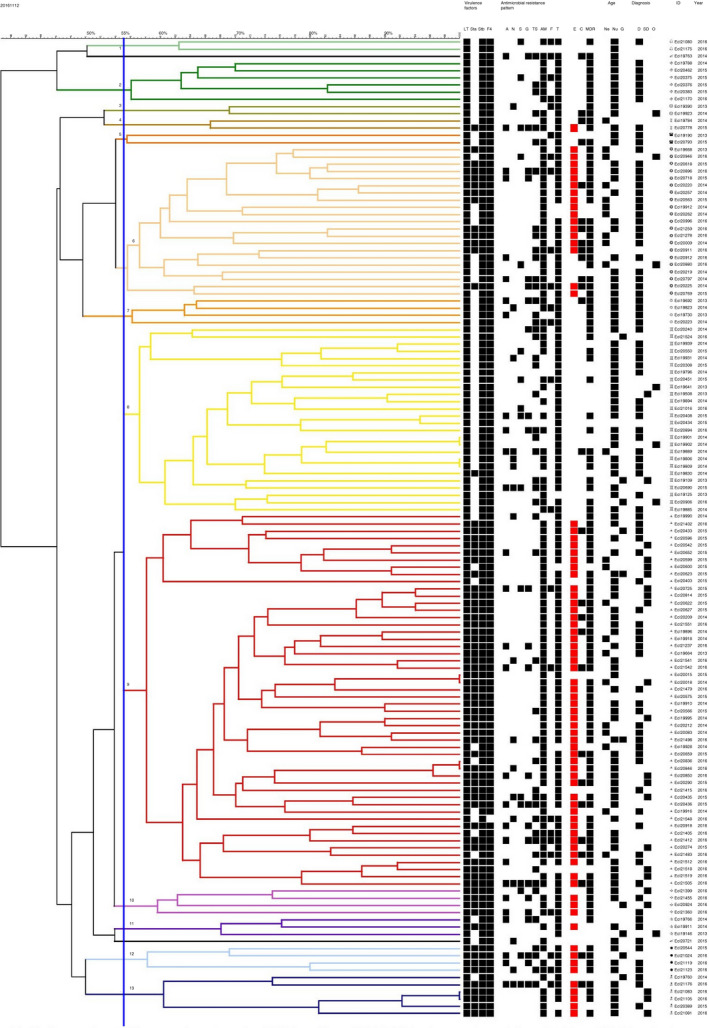
Phylogenetic additive tree based on the PFGE profiles of ETEC:F4 isolates collected from 2013 to 2016 by the EcL in diseased pigs in Quebec. D = Diarrhoea, SD = Sudden Death, O = Other, Ne = Neonates, Nu = Nursery, G = Grower, ID = Identification, A = Apramycin, N = Neomycin, S = Spectinomycin, G = Gentamicin, TM = Trimethoprim Sulfisoxazole, AM = Ampicillin, T = Tetracycline, F = Florfenicol, E = Enrofloxacin (the squares are coloured in red), C = Ceftiofur, MDR = Multidrug resistance.

A total of 137 isolates from 128 cases were typed by PFGE. They were grouped into 13 different clusters based on the PFGE profile as illustrated in Fig. 7. Two isolates with <50% of similarity were assigned to the cluster 0 (in black).

The cluster 8 (yellow) was mainly composed of LT:STb:F4 isolates susceptible to enrofloxacin whereas the cluster 9 mostly included LT:STb:STa:F4 isolates non‐susceptible to enrofloxacin. Together, they included 78/137 of the isolates. The cluster 6 (pale orange) is also of interest as it includes mainly (17/21) non‐susceptible to enrofloxacin isolates.

**Figure 8:**
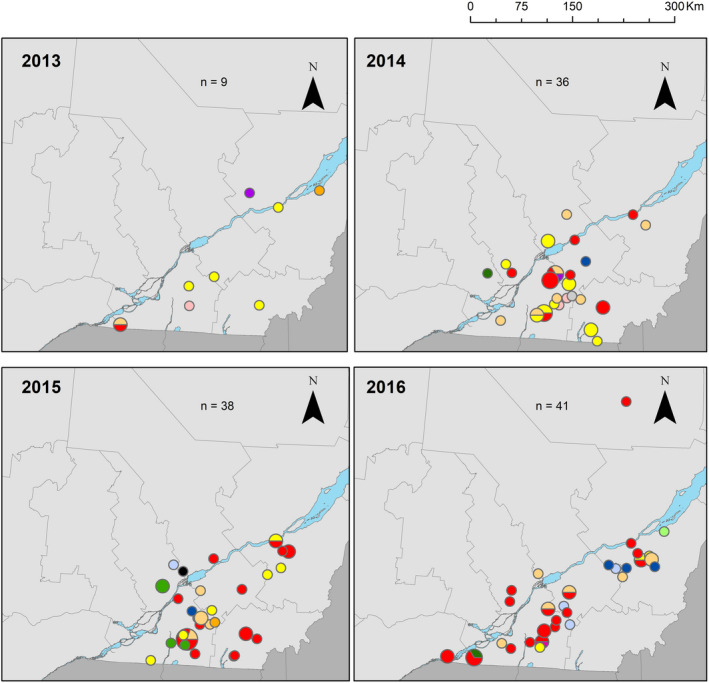
Geographical distribution of isolates belonging to the PFGE clusters over the Quebec territory in 2013–2016 according to the EcL database. The size of the circles is proportional to the number of isolates identified in the 6‐digit zip code region ( 1 isolate; 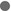 5 isolates; 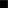 cluster 0; 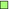 cluster 1; 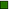 cluster 2; 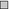 cluster 3; 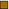 cluster 4; 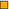 cluster 5; 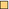 cluster 6; 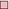 cluster 7; 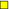 cluster 8; 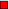 cluster 9; 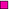 cluster 10; 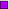 cluster 11; 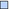 cluster 12; 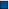 cluster 13).

As illustrated in Figs 7 and 8, isolates sampled in 2013 belonged mostly to cluster 8 (4/11 belonged to cluster 8, the 7 other isolates were dispatched in 6 other clusters). Only one isolate sampled in 2013 belonged to cluster 9. Among isolates sampled in 2015 and 2016, only 6/41 and 4/46, respectively, belonged to cluster 8, whereas many more belonged to cluster 9 (20/41 and 19/46 respectively). Geographical distribution of clusters 6 and 9 extended over the study period (Fig. 8).

The isolates of cluster 9 were found in both neonatal and nursery pigs, whereas those of group 8, except for one isolate, were found in nursery or grower pigs (Figs 7 and 8).

Out of 52 isolates in cluster 9, 46 manifested non‐susceptibility to enrofloxacin, and of these 46, 11 isolates manifested both ceftiofur and enrofloxacin resistance.

## Discussion

The main objective of this study was to describe the patterns of pathovirotypes and antimicrobial resistance of *E. coli* isolated from diseased pigs in Quebec from 2008 to 2016, using the APZEC database. Although it was predictable that ETEC:F4 would predominate as one of the main pathovirotypes isolated from cases of diarrhoea in pigs (Dubreuil *et al*. 2016), our results are notable as they highlight unique trends in Quebec. Indeed, in this province, ETEC:STEC:F18 and ETEC:F18 were not frequently identified, during this period of time, in cases of diarrhoea in pigs. These data contrast with those of most other countries, where these F18 pathovirotypes are equally or more prevalent than ETEC:F4, illustrating geographical variations (Luppi *et al*. 2016; Bessone *et al*. 2017; Van Breda *et al*. 2017; Do *et al*. 2019; Yang *et al*. 2019). STa:STb:F4, which has been found in sick pigs in some European countries (Luppi *et al*. 2016), appeared in Quebec only in 2014 and its frequency seems to have increased subsequently.

The presence of fimbriae‐negative EPEC, ETEC and STEC, in more than half of pathologic cases, is notable. The possibility of false negative results for F4 (other fimbriae are tested in single PCR) due to multiplex PCR is unlikely, as the sensitivity of this test has been calculated to be 99·1% when compared with whole genome sequencing results regarding the presence of F4 fimbriae (data not shown). Although it is difficult to evaluate their clinical significance, it is possible that certain ETEC and STEC carry fimbriae that are not tested routinely. Considering the fast evolution of the *E. coli* genome (Blount 2015), it is conceivable that new fimbrial genes have appeared. However, it would not be realistic (logistically and financially) to expect the detection of recently discovered genes in a diagnostic context. It is also conceivable that possible pathogenic pathotypes are opportunists or secondary pathogens, contributing to the development of disease in certain circumstances such as the presence of co‐infection or other predisposing factors.

We observed an increase in the number of cases positive for LT:STb:STa:F4 isolates non‐susceptible to enrofloxacin in 2013, which prevailed until the end of the study period and which was confirmed by the spatio‐temporal cluster of such cases between April 2015 and September 2016. Likewise, an increase in fluoroquinolone non‐susceptibility has also been described in pigs in Australia (Abraham *et al*. 2015), in the United States of America (Jiang *et al*. 2019) and especially in Japan (Kusumoto *et al*. 2016) where clusters of ETEC/STEC non‐susceptible to fluoroquinolones (based on the PFGE profile) have been recently reported to have emerged in pigs. Similarly, *E. coli* ST131, appeared concomitantly at several places in the world when it first emerged in the mid‐2000s, and has subsequently spread widely (Mathers *et al*. 2015). Non‐susceptibility to enrofloxacin might be one of the characteristics which confer to these isolates a selective advantage compared to the other isolates (Fuzi *et al*. 2020). Data on quinolone use in pigs in Canada are scarce, indeed, in the Canadian Integrated Program for Antimicrobial Resistance surveillance (CIPARS) report, fluoroquinolone use is surprisingly reported as inexistent, although these data concern only grower‐finisher pigs and do not provide information on use in farrowing barns or nurseries (Government of Canada 2016). However, it has been used off‐label in Quebec since the 2000s to treat ND and PWD. This emergence of resistance to enrofloxacin is of great concern as fluoroquinolones are now classified as critical antimicrobials (Health Canada 2009; World Health Organization 2019).

The number of isolates resistant to ampicillin and tetracycline were consistently high over the years and the number of MDR isolates increased over the study period. This is consistent with the results of previous study (Jahanbakhsh *et al*. 2015), and it can be explained by the predominant role that penicillins and tetracyclines have in porcine industry in Canada to treat diseased pigs and the extensive use of chlortetracycline prophylactically (Kim *et al*. 2005; Glass‐Kaastra *et al*. 2013).

In this study, we evaluated the phylogenetic relationship of ETEC:F4 isolates based on their PFGE profiles. We confirmed that the LT:STb:STa:F4 isolates are part of a cluster with 55% of similarity based on their PFGE profile. Isolates from this cluster seem to have emerged and spread through the Quebec territory in 2015 and 2016. This cluster also comprises some LT:STb:F4 non‐susceptible isolates, collected in 2015 and 2016, suggesting that the *estA* gene may have been acquired horizontally and lost by certain isolates; however, the change in the PFGE profiles should be minimal with the loss of one plasmid (Barton *et al*. 1995). The other possibility would be the concomitant emergence of another, closely related LT:STb:F4 strain. It should be noted that phylogenetic trees built on PFGE profiles with the UPGMA method are more reliable when isolates are minimally divergent (Hillis *et al*. 1994). We performed this analysis to assess the possible presence of a ‘high‐risk’ clone, similar to the human ExPEC *E. coli* ST131. In contrast, the ETEC:F4 isolates were relatively diverse according to their PFGE profile, which, at first sight, does not support the ‘high‐risk’ clone hypothesis but suggests that virulence, fitness and antimicrobial resistance genes could be transferred through horizontal gene transfer (HGT). However, despite its relative unreliability due to HGT events and subjectivity (Price *et al*. 2013), this approach has proven useful in the past, even when the Tenover criteria for relatedness (Tenover *et al*. 1995) are not fulfilled. For example, it seems that Woodford et al. had already identified isolates from the ‘high‐risk’ clone *E. coli* ST131 as early as 2004 (Woodford *et al*. 2004). This phylogenetic group was not recognized as such before 2008 (Lau *et al*. 2008), because based on phylogenetic relationships using PFGE, it only demonstrated 60% similarity. Although isolates for the spatiotemporal cluster and isolates belonging to the PFGE cluster 9 were not selected in the same way and therefore cannot be compared, it is noticeable that in both analyses, isolates non‐susceptible to enrofloxacin gained in importance in 2015.

The detection of a spatiotemporal cluster is noteworthy. The underlying reasons for the center of the Montérégie region having the highest incidence of cases positive for LT:STb:STa:F4 non‐susceptible to enrofloxacin would need further investigation. The high density of swine farms, direct and indirect contacts between farms through pig movements and people (such as veterinarians, transporters, etc.) visiting these farms and possibly antimicrobial prescriptions habit in this region are potential factors that could facilitate the dissemination or emergence of resistant isolates, but were not investigated in this study. After 2016, the spatio‐temporal cluster was no longer detected, which does not mean that enrofloxacin non‐susceptibility has disappeared but more likely that it has extended to other virotypes, particularly the LT:STb:F4 and to other geographical areas.

The main limitation of our study is that we have based our results on passive surveillance. The increase in LT:STb:STa:F4 isolate detection could have been biased by an increased of submissions of cases to the EcL due to the fact that we informed field veterinarians very early of the occurrence of enrofloxacin non‐susceptibility. This may have encouraged veterinarians to submit more cases or some specific cases to detect antimicrobial resistance. For the spatiotemporal analysis, this bias was likely minimized by the fact that we used control isolates from the same database during the same period.

Our results reinforce the importance of regional surveillance programmes to generate relevant data on the locally present pathovirotypes and virotypes, which is essential for the development of specific detection or prevention strategies, such as new diagnostic tests or specific vaccines. They also underline the importance of accurate identification of antimicrobial patterns associated with disease, to ensure the instigation of the most appropriate treatment (antimicrobial therapy) and prevention strategy (such as vaccines). To this purpose, the EcL developed the APZEC database. This platform primarily reports the presence of virulence genes, as detected by PCR, and antimicrobial resistance on a case based and isolate based level to highlight the temporal and spatial trends and allow correlations with the demographic data. Therefore, the APZEC database has been very useful to identify and monitor an emerging virotype/cluster. A potential improvement of this tool would be to integrate additional information such as the antimicrobial use in a systematic and standardized way.

In conclusion, the APZEC database has allowed the description of the temporal trends of *E. coli* pathotypes detected from diseased pigs, in Quebec, between 2008 and 2016. We highlighted the predominance of the ETEC:F4 pathovirotype, the emergence of the LT:STb:STa:F4 virotype and the occurrence of fluoroquinolone non‐susceptibility. We have also identified a PFGE cluster composed mainly of LT:STb:STa:F4 ETEC isolates non‐susceptible to enrofloxacin, in the porcine population in Quebec in 2015. Further investigation, especially on molecular mechanisms of resistance to fluoroquinolones, are needed to determine the risk it might represent for porcine and public health. We consider the APZEC database to be a very powerful tool providing a framework for diagnostic and research laboratories to work together to improve animal health.

## Conflict of Interest

The authors of this manuscript declare no conflict of interest.

## Supporting information


**Figure S1**. Susceptibility to antimicrobials in ETEC:F4 isolates from positive cases, detected in samples from diseased pigs of Quebec submitted to the EcL from 2008 to 2016.

**Table S1**. Frequency of clinical signs in 3773 cases of diseased pigs in Quebec submitted to the EcL from 2008 to 2016. (when several clinical signs were present, the most severe one was attributed to the case).

**Table S2**. Complete profile of all isolates entered in the APZEC database between 2008 and 2016. Missing data are represented by ******
