# Supplementary material for: A new multidrug‐resistant enterotoxigenic Escherichia coli pulsed‐field gel electrophoresis cluster associated with enrofloxacin non‐susceptibility in diseased pigs
Source: J Appl Microbiol. 2020 Aug 25;130(3):707–21. doi: 10.1111/jam.14816 (PMC7984379; doi:10.1111/jam.14816)
Supplement: Supplementary file 1 — Figure S1. Susceptibility to antimicrobials in ETEC:F4 isolates from positive cases, detected in samples from diseased pigs of Quebec submitted to the EcL from 2008 to 2016. [file JAM-130-707-s001.pdf]

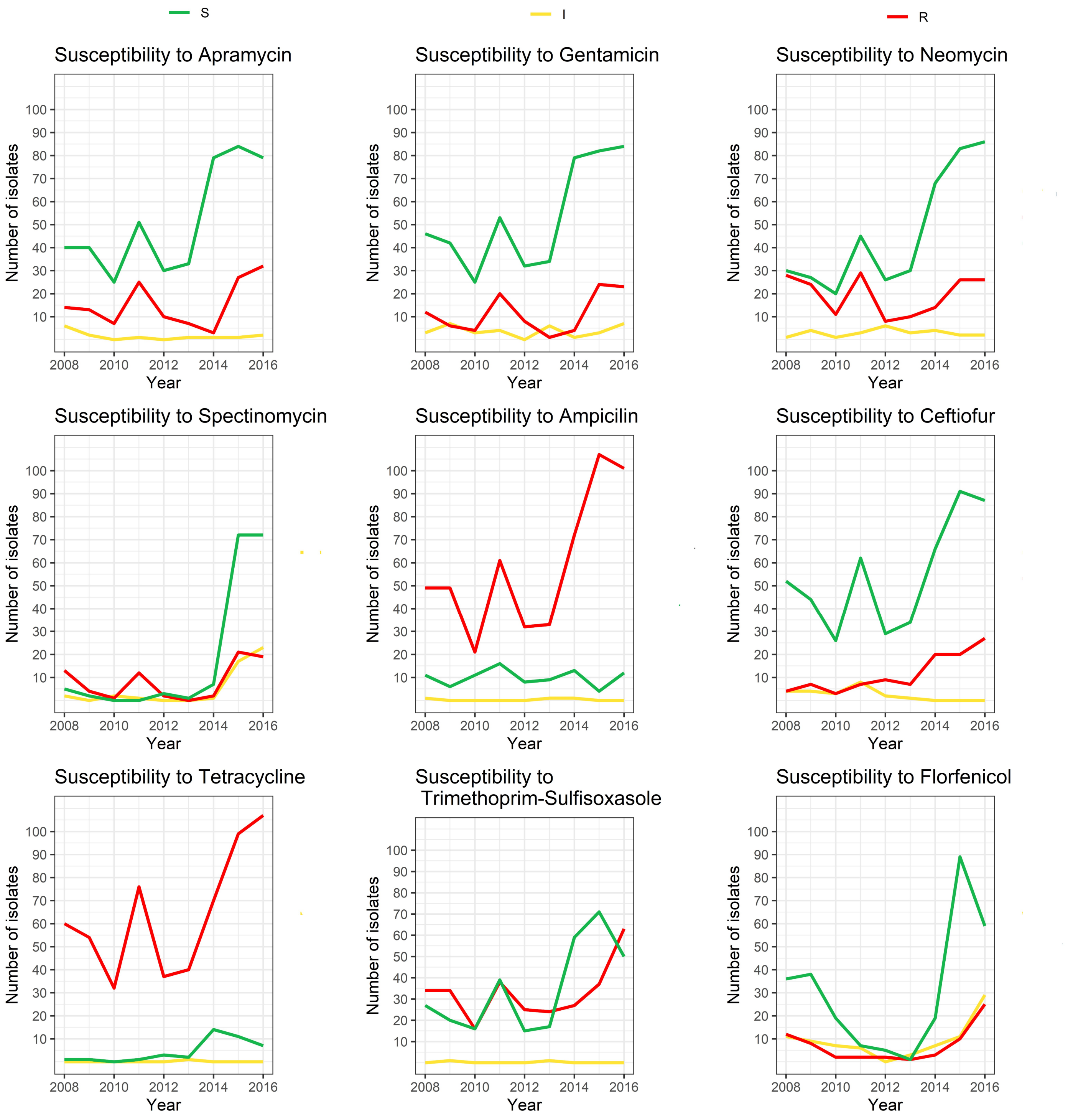

Figure S1: Susceptibility to antimicrobials in ETEC:F4 isolates from positive cases, detected in samples from diseased pigs of Quebec submitted to the EcL from 2008 to 2016.
