# Supplementary material for: A new multidrug‐resistant enterotoxigenic Escherichia coli pulsed‐field gel electrophoresis cluster associated with enrofloxacin non‐susceptibility in diseased pigs
Source: J Appl Microbiol. 2020 Aug 25;130(3):707–21. doi: 10.1111/jam.14816 (PMC7984379; doi:10.1111/jam.14816)
Supplement: Supplementary file 2 — Table S1. Frequency of clinical signs in 3773 cases of diseased pigs in Quebec submitted to the EcL from 2008 to 2016. (when several clinical signs were present, the most severe one was attributed to the case). [file JAM-130-707-s003.docx]

Table S1 : Frequency of clinical signs in 3773 cases of diseased pigs in Quebec submitted to the EcL from 2008 to 2016. (when several clinical signs were present, the most severe one was attributed to the case).

| Clinical signs | Number of cases (% ) |
| --- | --- |
| Diarrhea | 1661 (44) |
| Sudden death | 490 (13) |
| Edema | 188 (5) |
| Wasting | 189 (5) |
| Respiratory symptoms | 189 (5) |
| Other (abortion, septicemia, nervous symptoms, urinary symptoms) | 151 (4) |
| Unknown | 905 (24) |
